# Investigation of Microbial Translocation, *TLR* and *VDR* Gene Polymorphisms, and Recurrence Risk in Stage III Colorectal Cancer Patients

**DOI:** 10.3390/cancers14184407

**Published:** 2022-09-10

**Authors:** Ippokratis Messaritakis, Asimina Koulouridi, Eleni Boukla, Maria Sfakianaki, Konstantinos Vogiatzoglou, Michaela Karagianni, Nikolaos Gouvas, John Tsiaoussis, Evangelos Xynos, Elias Athanasakis, Dimitrios Mavroudis, Maria Tzardi, John Souglakos

**Affiliations:** 1 Laboratory of Translational Oncology, Medical School, University of Crete, 70013 Heraklion, Greece; 2 Medical School, University of Cyprus, 20537 Nicosia, Cyprus; 3 Department of Anatomy, School of Medicine, University of Crete, 70013 Heraklion, Greece; 4 Department of Surgery, Creta Interclinic Hospital of Heraklion, 71305 Heraklion, Greece; 5 Department of General Surgery, Heraklion University Hospital, 71100 Heraklion, Greece; 6 Department of Medical Oncology, University General Hospital of Heraklion, 71100 Heraklion, Greece; 7 Laboratory of Pathology, Medical School, University of Crete, 70013 Heraklion, Greece

**Keywords:** colorectal cancer, CRC, microbial translocation, *VDR*, polymorphisms, genetic variants, dysbiosis, *TLR*

## Abstract

**Simple Summary:**

Gut microbial dysbiosis and microbial passage into the peripheral blood leads to colorectal cancer (CRC) and disease progression. Toll-like (*TLR*) and vitamin D (*VDR*) receptors play important role in the immune modulation and polymorphisms that may increase CRC risk. The aim of the current study was to demonstrate the prognostic value of microbial DNA fragments in the blood of stage III CRC patients and correlate such microbial detection to *TLR*/*VDR* polymorphisms. *TLR*/*VDR* polymorphisms and presence of microbial DNA in CRC patients highlight their role in cancer development and progression.

**Abstract:**

Gut microbial dysbiosis and microbial passage into the peripheral blood leads to colorectal cancer (CRC) and disease progression. Toll-like (*TLR*) and vitamin D (*VDR*) receptors play important role in the immune modulation and polymorphisms that may increase CRC risk and death rates. The aim of the current study was to demonstrate the prognostic value of microbial DNA fragments in the blood of stage III CRC patients and correlate such microbial detection to *TLR/VDR* polymorphisms. Peripheral blood was collected from 132 patients for the detection of microbial DNA fragments, and *TLR*/*VDR* gene polymorphisms. In the detection of various microbial DNA fragments, *TLR* and *VDR* polymorphisms was significantly higher compared to healthy group. Homozygous individuals of either *TLR* or *VDR* polymorphisms had significantly higher detection rates of microbial DNA fragments. Mutational and MSI status were significantly correlated with *TLR9* and *VDR* polymorphisms. Significantly shorter disease-free survival was associated with patients with *BRAF* mutated tumors and *Apa*I polymorphisms, whereas shorter overall survival was associated with the detection of *C. albicans*. The detection of B. fragilis, as demonstrated by the multivariate analysis, is an independent poor prognostic factor for shorter disease-free survival. *TLR/VDR* genetic variants were significantly correlated with the detection of microbial fragments in the blood, and this in turn is significantly associated with tumorigenesis and disease progression.

## 1. Introduction

Although screening and new treatment strategies have been established for colorectal cancer (CRC), it remains a major health issue [1]. CRC is the third and second most common cancer in men and women, respectively [1,2]. Stage III CRC patients have an almost 60% 5-year overall survival rate and an almost 80% disease free survival rate [3].

CRC tumorigenesis, growth, and metastasis is a complex process, which involves molecular changes, microbial dysbiosis, impaired immunity, changes in stroma, etc. Transcriptomic profiling, microsatellite’s instability, mutation’s characteristics, somatic copy alternations number, and DNA methylation have been used as criteria for this categorization [4]. All these characteristics interact with each other and are influenced by microbial homeostasis, immune activation, or other parameters that interfere with tumorigenesis.

Microbial dysbiosis has been proven as a factor of tumorigenesis and tumor growth, especially in CRC [5,6]. Its interference with the immune system is already known [7]. Major parts of innate immunity are covered by the pattern recognition receptors (PRRs), expressed by dendritic cells and macrophages [8]. PRRs consist of Toll-like (*TLR*s) and NOD-like receptors. *TLR*s are transmembrane receptors that can detect any possible pathogens by recognizing and binding to pathogen-associated molecular patterns (PAMPs) [9]. This leads to an activation of inflammatory pathways. Their role seems to be dual in tumorigenesis [10]. *TLR*s 3, 5, 7, 8, and 9 enhance anti-tumor immunity through type I interferon, while *TLR*s 2 and 4 play a tumor-promoting role through NF-κΒ activation [11,12,13].

In recent years, the protective role of vitamin D in carcinogenesis or tumor growth is under evaluation [14,15]. Its action, especially of calcitriol, is partially regulated by the vitamin D receptors (*VDR*s), which are in abundance in the intestinal epithelium [16]. Palmer et al., showed that *VDR* expression in SW 480-ADH cells is suppressed by SNAI1 and SNAI2 transcription factors [17]. Human CRC cells have high levels of RNA encoding SNAI1 and SNAI2, leading to *VDR*s downregulation and interruption of vitamin D antitumor effect [17,18]. Vitamin D antitumor effect mainly interferes with proliferation, differentiation, apoptosis, angiogenesis, and immune regulation [19].

As we have previously showed, specific polymorphisms of *VDR*s are strongly correlated with tumorigenesis, tumor growth, and poor prognosis at stage II–IV CRC patients, the same as with *TLR*s polymorphisms [20,21]. *TLR*s polymorphisms were significant higher at CRC cancer patients than the control group and were correlated with stage IV disease and shorter overall survival [20]. Moreover, microbial translocation has been correlated to CRC tumorigenesis, metastatic disease, and shorter survival at a relevant patients group [6]. All these highlight a strong correlation between the detection of specific *VDR* polymorphisms, *TLR*s specific genetic variants, and microbial translocation, as well as the complexity of CRC. In the current study, we aimed to detect and evaluate the role of specific *VDR*s and *TLR*s polymorphisms, along with microbial translocation, in the recurrence risk of stage III CRC patients.

## 2. Materials and Methods

### 2.1. Patients and Healthy Controls Enrollment

Since August 2018 to June 2022, a total of 132 consecutive patients from the Department of Medical Oncology, University Hospital of Heraklion, aged >18 years old, with newly diagnosed and with histologically documented stage III CRC were enrolled in the study. None of the patients had history of other malignancy. Moreover, a total of 100 healthy individuals, aged >18 years old were also enrolled in the study.

### 2.2. Blood and Primary Tissue Samples

Peripheral blood (5 mL in EDTA) was collected from all patients and control subjects enrolled in the study, and the QIAamp DNA Blood Mini Kit (QIAGEN, Hilden, Germany) was used for DNA extraction. Concerning the primary tissues, for microdissection of representative formalin fixed paraffin embedded (FFPE) specimens, a piezoelectric microdissector (Eppendorf, Hamburg, Germany) was used to collect cancer cells [22], following evaluation of the appropriate area by an experienced pathologist. DNA extraction was performed using both the MasterPure^™^ Complete DNA and RNA Purification Kit (Epicenter, Madison, WI, USA) following the manufacturer’s instructions. NanoDrop ND-1000 v3.3 (Thermo Fisher Scientific, Wilmington, DE, USA) was used for DNA quantification.

### 2.3. Microbial DNA Amplification

For the microbial DNA amplification, each genes’ target reagents and PCR conditions used have already been covered in detail by our team [6,23]. In brief, a set four primer pairs were used for the detection of bacterial genomic DNA encoding 16S rRNA, glutamine synthase of *Bacteroides fragilis*, *β*-galactosidase gene of *Escherichia coli,* and 5.8S rRNA found in *Candida* *albicans*. The samples’ DNA integrity was checked using the reference gene human glyceraldehyde phosphor-dehydrogenase (GAPDH). In order to identify bacterial DNA in blood samples, 16S rRNA was employed as a reference.

### 2.4. Toll-Like Receptor (TLR) and Vitamin D Receptor (VDR) Genotyping

For genotyping of *TLR* genetic variants, polymerase chain reaction and restriction fragment length polymorphism methodologies (PCR-RFLP) were performed. In brief, *TLR2* 196-to-174 Ins/Del genetic variants were determined by PCR, whereas *TLR4 *(Asp299Gly and Thr399Ile) and *TLR9 *(T1237 and T1486C) genetic variants were determined by PCR-RFLP. All materials and conditions for each gene target involved in the current study have already been described previously by others and our group [20,21,24].

For the genotyping of *VDR* genetic variants, as in the case of *TLRs*, PCR-RFLP methodology was performed. Each genes’ target reagents and PCR conditions used have already been covered in detail by our team [20,21].

In brief, for all single nucleotide polymorphisms (SNPs) of the *TLR* gene or *VDR* gene at the *Taq*I, *Apa*I, *Fok*I, and *Bsm*I positions, the patients were characterized as wild type, heterozygous, or homozygous in the absence of the restriction site in both alleles, the presence of the restriction site in one allele, and presence of the restriction site in both alleles, respectively.

### 2.5. Mutational Analysis

*KRAS*, *NRAS,* and *BRAF* mutational analyses were performed by Sanger sequencing, following amplification of *KRAS* exon 2, 3, and 4; *NRAS* exon 2, 3, and 4; and *BRAF* exon 15. Moreover, microsatellite instability (MSI) status was also evaluated. All materials and conditions for each gene target involved in the current study have already been described previously by our group [25,26,27,28].

## 3. Results

### 3.1. Patients and Healthy Donors Characteristics

**Figure 1:**
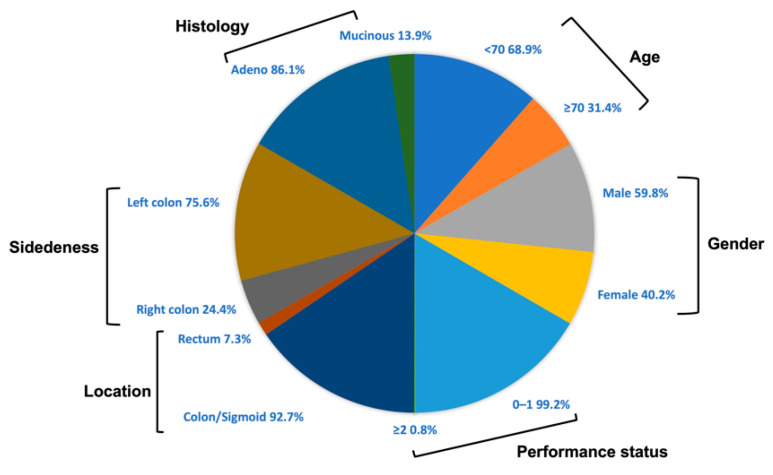
Patients’ characteristics.

In total, 132 stage III CRC patients and 100 healthy donors have been enrolled in this single-centered study. The median age of the patients and healthy subjects enrolled was 62 years (range: 36–83 years) and 66 years (range: 35–89 years), respectively. Most of them were males (patients: 59.1%; healthy donors: 54%) and <70 years old (patients: 68.9%; healthy donors: 70%). Most of the patients had a good performance status (PS) (99.2%) and had left sided tumors (75.8%), mainly on sigmoid (50.7%). All patients were diagnosed with adenocarcinoma, and mucinous features were observed in 18 (13.9%) patients, who received CAPOX (capecitabine + oxaliplatin) as an adjuvant treatment (55.7%). The patients’ characteristics and demographics are demonstrated in Figure 1 and Table 1 and Table S1.

### 3.2. Detection of Microbial DNA Fragments

For both CRC patients and healthy subjects, the detection of microbial DNA fragments in the peripheral blood was evaluated. The detection of microbial fragments for 16S rRNA, *E. coli*, *B. fragilis,* and *C. albicans* was demonstrated in 57 (43.2%), 27 (20.5%), 42 (31.8%), and 48 (36.4%), respectively, in CRC patients, and in 16 (16%), 16 (16%), 0 (0%) and 0 (0%), respectively, in healthy controls (Table 2 and Table S1). A significant difference was demonstrated in all cases of microbial DNA fragments (*p* < 0.001) except for the *β*-galactosidase gene of *E. coli (p* = 0.387), between CRC patients and healthy controls (Table 2 and Table S1).

### 3.3. TLR and VDR Genetic Variants Analysis and Clinical Outcoume

All CRC patients and healthy subjects were also evaluated for the presence of genetic variants in both *VDR* and *TLR* genes. For the case of *VDR* genetic variants, 20 (15.2%), 49 (37.1%), 20 (15.2%), and 17 (12.9%) patients presented the homozygous mutant genotype for *Taq*I, *Apa*I, *Fok*I, and *Bsm*I polymorphisms, respectively (Table 3 and Table S1). A significant difference was demonstrated for all *VDR* gene polymorphisms between CRC patients and healthy donors (*p* < 0.001) (Table 3 and Table S1). Moreover, 51 (38.6%), 52 (39.4%), 49 (37.1%), 47 (35.6%), and 75 (56.8%) patients presented the homozygous mutant genotype for *TLR4*—D299G, *TLR4*—T399I, *TLR9*—T1237C, *TLR9*—T1486C, and *TLR2*-196 to -174bp, respectively (Table 3 and Table S1). None of the healthy subjects presented homozygous (for all 5 *TLR* polymorphisms evaluated) or even heterozygous (for *TLR2* and *TLR4*) mutant genotypes. A significant difference was also demonstrated for all *TLR* gene polymorphisms between CRC patients and healthy donors (*p* < 0.001) (Table 3 and Table S1).

**Figure 2:**
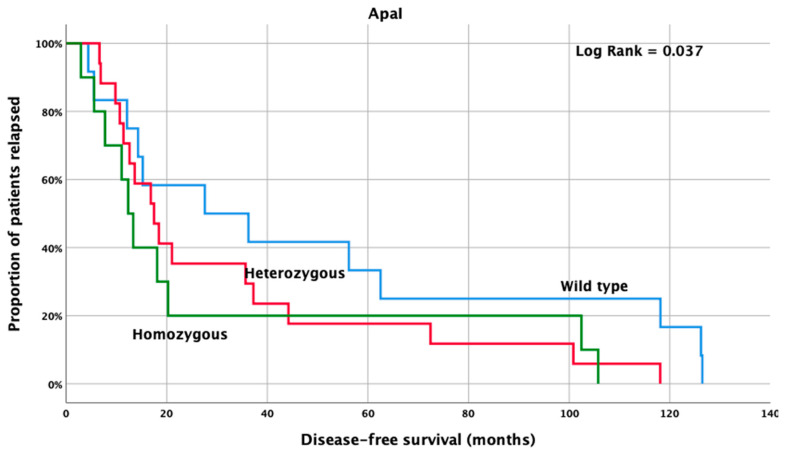
Kaplan Meier curve for disease-free survival according to *VDR*-*Apa*I genetic variants, in stage III CRC patients.

Among CRC patients, the homozygous mutant *Apa*I genetic variants presented with significantly lower DFS (12.3 months, 95% CI: 8.8–15.9 months) when compared to heterozygous and wild type patients (17.5 months, 95% CI: 11.0–23.9 months and 27.6 months, 95% CI: 0.0–63.2 months; *p* = 0.036) (Figure 2).

### 3.4. Correlation of Microbial DNA Fragments with TLR and VDR Genetic Variants Analysis

The correlation of the presence of microbial DNA fragments, *TLR*, and *VDR* genetic variants in the peripheral blood of CRC patients were investigated (Table 4). As it was demonstrated, a significant coexistence was shown between the detection of 16S rRNA and *TLR4* (D299G and T399I), *Taq*I and *Fok*I polymorphisms (*p* = 0.009; *p* = 0.043; *p* < 0.001 and *p* < 0.001, respectively); *E.coli* was significantly associated with *Taq*I and *Apa*I polymorphisms (*p* < 0.001 and *p* = 0.003, respectively); *B. fragilis* was significantly associated with *TLR4* (D299G) *Taq*I, *Apa*I, and *Fok*I polymorphisms (*p* = 0.025; *p* < 0.001; *p* = 0.009 and *p* < 0.001, respectively); whereas *C. albicans* was significantly associated with *Taq*I, *Apa*I, *Fok*I and *Bsm*I polymorphisms (*p* < 0.001; *p* = 0.015; *p* = 0.027 and *p* = 0.029, respectively). Moreover, a significant association was demonstrated among the existence of almost all different *TLR* and *VDR* genetic variants (Table 4). Additionally, *TLR2*-196 to -174bp was significantly associated with *Bsm*I (*p* = 0.004); *TLR4*—D299G was significantly associated with *Taq*I, *Apa*I, *Fok*I, and *Bsm*I (*p* = 0.043; *p* < 0.001; *p* = 0.015 and *p* < 0.001, respectively); *TLR4*—T399I was significantly associated with *Taq*I, *Apa*I, *Fok*I, and *Bsm*I (*p* = 0.042; *p* < 0.001; *p* = 0.036 and *p* < 0.001, respectively); *TLR9*—T1237C was significantly associated with *Apa*I, *Fok*I, and *Bsm*I (*p* = 0.008; *p* = 0.012 and *p* < 0.001, respectively) and *TLR9*—T1486C was significantly associated with *Apa*I, *Fok*I, and *Bsm*I (*p* = 0.018; *p* = 0.029 and *p* < 0.001, respectively).

### 3.5. Association of Tumor Mutations and MSI Status with Microbial DNA Fragments, TLR and VDR Polymorphisms

As it is demonstrated in Table 5, the association of *TLR* and *VDR* genetic variants with *RAS*, *RAF,* and MSI status was also investigated. Of the 132 enrolled patients, *KRAS*, *NRAS*, *BRAF^V600E^*, and MSI status was available in 62 (47%), 57 (43.2%), 54 (40.9%), and 62 (47%) CRC patients, respectively (Table S1). Of those, 25 (40.3%), 1 (1.8%), 5 (9.3%), and 7 (11.3%) were *KRAS*, *NRAS,* and *BRAF^V600E^* mutants and MSI-High, respectively (Table S1). As it was observed, KRAS mutations were significantly associated with *TLR9*—T1237C (*p* = 0.014) and *TLR9*—T1486C (*p* = 0.006) polymorphisms and *BRAF^V600E^* mutations were significantly associated with *TLR9*—T1486C (*p* = 0.045) polymorphisms, whereas MSI-High status was significantly associated with *TLR9*—T1237C (*p* = 0.012), *Taq*I (*p* = 0.025), *Apa*I (*p* = 0.047), *Fok*I (*p* = 0.001), and *Bsm*I (*p* < 0.001).

### 3.6. Univariate and Multivariate Analysis for Cox Regression Analysis

According to the Cox regression univariate analysis, *BRAF^V600E^* mutations, histology (adenocarcinoma vs. mucinous), the detection of microbial DNA encoding for glutamine synthase of *B. fragilis*, and the detection of *Apa*I mutant alleles of the *VDR* gene are significantly associated with a shorter disease-free survival (DFS); and the detection of microbial DNA encoding for 5.8S rRNA is significantly associated with shorter overall survival (OS) (Table 6). Based on the Cox regression multivariate analysis, adjusting for above mentioned factors, *B. fragilis* is a significant independent factor linked to shorter OS (HR: 33.85, 95% CI: 1.8–622.4, *p* = 0.018) (Table 6).

## 4. Discussion

Different pathways have been under research to understand tumorigenesis and develop new treatments against CRC. *TLR*s, microbiota, and *VDR*s are some of the areas that are under evaluation, involved in pathways that modulate immunity against cancer. In the current research, we aimed to evaluate the existence of *TLR*s and *VDR*s polymorphisms and of microbial translocation and their correlation with prognosis, in stage III CRC patients.

The passage of intestinal microorganisms into the bloodstream (also known as microbial translocation) is a phenomenon mainly met because of microbial dysbiosis [23]. This disturbance in microbial composition has been proven as one of the ways of tumorigenesis and tumor growth in CRC [6,29]. The microbiota that has escaped to the blood stream is detected mainly by its fragments or its products [6]. As our group has previously demonstrated, 16SrRNA, *E. coli*, B. fragilis, and 5.8S rRNA microbial fragments detected are correlated with tumorigenesis and progression and may have a prognostic role in CRC patients [6]. This study confirms the previous results, focusing on stage III CRC patients. Numerically, all DNA fragments were more frequently detected in CRC patients than in the healthy donors and this detection was statistically significant, except in the case of the detection of *β*-galactosidase gene of *E. coli.*

*TLR*s are a part of innate immunity, contributing mainly to the recognition of external factors that could be pathogens [10]. MyD88-dependent pathway plays a crucial role in the immune reaction and CRC related to inflammation [7,10]. Previous studies have shown that different polymorphisms have been detected in CRC patients. *TLR* 3/4/7/8/9 and their polymorphisms seem to have a prognostic role for CRC [20,30]. Specifically, high expression of *TLR4*-mediated MyD88 signaling has been correlated with poor prognosis, even in stroma and CAFs related to CRC [31,32]. Also, *TLR4* along with *TLR2* and *TLR3* may have a prognostic role for CRC through regulation of NFκΒ pathway, leading to tumorigenesis [33]. *TLR7* and *TLR8* expressed by CD133^+^ cells have been linked to worse prognosis in CRC patients [34]. On the other hand, there are some controversial results concerning *TLR*s and their prognosis in various tumor types [35,36]. Specifically for CRC, high expression of *TLR5* in tissue seems to be linked with better prognosis [37]. On a previous study of our group, it was demonstrated that higher frequencies of *TLR2*, *TLR4*, and *TLR9* polymorphisms in CRC patients, in comparison to healthy individuals, are correlated with worst prognosis [20]. *TLR2*-196 to-174 del/del genotype, *TLR4* Asp299Gly, *TLR4* Thr399Ile, *TLR9* T1237C, and *TLR9* T1486C homozygous genotypes were all detected in statistically significant higher levels in the disease setting and were also correlated with worst prognosis [20]. All the above results are in accordance with the results of the current study; that is, higher rates of all *TLR* genetic variants detected in CRC patients compared to health individuals. Particularly for the homozygous mutant genotype for *TLR4*—D299G, *TLR4*—T399I, *TLR9*—T1237C, *TLR9*—T1486C, and *TLR2*-196 to-174 bp the detection was up to 56.8% in CRC patients, while none of healthy donors were detected with any of the variations. Nevertheless, no statistically significant prognostic value was demonstrated in stage III CRC patients.

The multiple roles vitamin D plays in carcinogenesis, protection, or therapy on CRC have been being researched over the last handful of decades [19]. Vitamin D fulfills its role by binding to its receptors, so *VDR* polymorphisms could affect the signaling pathway vitamin D activates [38]. More than 60 single nucleotide polymorphisms (SNPs) of *VDR* gene have been referred to in previous studies as related to carcinogenesis and prognosis at different tumor types [39]. The SNPs are found mainly in the promoter region in exons 2–9 and in the 3′-UTR (3′-untranslated regions) of the gene [40]. However, only some of them are directly related and functionally important in CRC. These include *Taq*I (rs731236; *Thermus aquaticus* I), *Apa*I (rs7975232; *Acetobacter pasteurianus* sub. *pasteurianus* I), *Bsm*I (rs1544410, *Bacillus stearothermophilu*s I), and *Fok*I (rs2228570; *Flavobacterium okeanokoites* I), located in exon 9, in the intron between exons 8 and 9 and in exon 2, respectively [41,42,43,44,45]. Previous studies have highlighted the role of *VDR*s in immune modulation mainly through regulation of gut microbiota and microbial translocation. *VDR* conditional knockout (*VDR*^ΔIEC^) in the epithelium of colon or low intestinal *VDR* protein levels may lead to microbial translocation [46]. Reduction of JAK/STAT (Janus kinases/signal transducer and activator of transcription proteins) signaling is another form of interference with gut microbiota and inflammatory responses [47]. Also, vitamin D has a promoting role for *TLR*s through binding to *VDR*s, leading to activation of innate immunity and modulation of gut microorganisms [48,49]. Different studies have tried to evaluate the role of *VDR* polymorphisms in CRC, with many controversial results, possibly because of the investigation of different populations [50,51,52]. Nevertheless, meta-analyses and reviews have shown a significantly higher level of detection of the homozygous mutant genotypes of *Taq*I and *Apa*I genotypes in CRC patients [53], whereas CRC tumorigenesis was correlated strongly with *Bsm*I, *Fok*I, and *Taq*I polymorphisms [54]. We have previously showed that the homozygous mutant genotypes of all *Taq*I, *Apa*I, *Bsm*I, and *Fok*I are significantly more frequent in CRC patients of all stages comparing to healthy donors [21]. However, the detection was in lower levels in early stages [21]. Herein, we validated the results above. Although the homozygosity of the polymorphisms were not so frequent, even in CRC patients, all evaluated polymorphisms were significantly more frequently detected in stage III CRC patients compared to the healthy population. This strongly suggests that *VDR* polymorphisms can contribute to CRC tumorigenesis. Regarding their correlation with *TLR* polymorphisms, almost all *VDR* and *TLR* polymorphisms were significantly correlated, promoting the hypothesis of their synergy. Correlating the detection of microbial fragments with *VDR* and *TLR* polymorphisms in stage III CRC patients, not all detected fragments were significantly correlated almost every polymorphism, and this is in contrast to our previous study. However, the correlation remains high, mainly for *VDR* polymorphisms and *TLR4—*D299G. All these results strengthen the hypothesis that *VDR* and *TLR* polymorphisms act together for the remodeling of gut microbiota and dysbiosis, disturbing the homeostasis and leading to immune modulation.

Finally, taking for granted the prognostic significance of tumor mutational status, *KRAS* and *BRAF* mutations were correlated with *TLR9—*T1237C, *TLR9—*T1486C polymorphisms, and *TLR9—*T1486C, respectively. MSI high status was correlated mainly with *TLR9—*T1237C and all *VDR* polymorphisms. Having in mind that *TLR9* participates in CRC tumorigenesis through inflammation, the results can be biologically reasonable [55,56,57].

To our knowledge, our research remains the only effort to investigate the detection and possible significance for CRC tumorigenesis and progression of microbial translocation, *TLR* and *VDR* polymorphisms, and their correlation in stage III CRC patients. This research is prospective, includes a homogeneous population, is well-distributed, and took into consideration important pathological features. However, limitations do exist. The sample is relatively small and the total time of follow up needs to be extended for safer prognostic results. Despite the limitations, *TLR* and *VDR* polymorphisms, as well as microbial translocation, seem to maintain an important role to CRC tumorigenesis and progression in stage III CRC patients and could modulate immune reaction to CRC. On that basis, more research remains to be done to validate these results and drive to new, multi-targeted preventive and therapeutic options.

## 5. Conclusions

In conclusion, our study remains the first attempt to evaluate the detection and possible prognostic significance of *TLR* and *VDR* polymorphisms and microbial translocation in the Greek population in stage III CRC patients. All polymorphisms were significantly more frequently detected in CRC patients than in healthy donors, and the same was observed for microbial fragments with the exception of the *β*-galactosidase gene of *E. coli*. Also, these parameters seem to correlate each other, empowering the hypothesis that immune modulation against CRC is a complex axis with possible multiple preventive and therapeutical targets.

## Figures and Tables

**Table 1:** Patients and healthy controls characteristics.

Demographics	Patients		Healthy Controls	
Characteristics	Frequency (N = 132)	%	Frequency (N = 100)	%
Age median (range)	62 (36–83)		65 (35–89)	
<70	91	68.9	70	70.0
≥70	41	31.1	30	30.0
Gender				
Male	78	59.1	54	54.0
Female	54	40.9	46	46.0
Performance status				
0–1	131	99.2		
≥2	1	0.8		
Location				
Colon/Sigmoid	122	92.4		
Rectum	10	7.6		
Right/Left site				
Right colon	32	24.2		
Left colon	100	75.8		
Histology				
Non-Mucinous	114	86.4		
Mucinous	18	13.9		
Regimen				
Folfox	59	44.3		
Capox	73	55.7		
Treatment Duration				
3 months	66	50.0		
6 months	66	50.0		
T status				
T2	7	5.3		
T3	95	72.0		
T4	30	22.7		
N status				
N0	22	16.7		
N1	81	61.4		
N2	29	21.9		
Microsatellite Instability (MSI)				
Stable	55	41.7		
High	7	5.3		
Unknown	70	53.0		
*KRAS*				
Wild type	37	41.7		
Mutant	25	5.3		
Unknown	70	53.0		
*NRAS*				
Wild type	56	42.4		
Mutant	1	0.8		
Unknown	75	56.8		
*BRAF*				
Wild type	49	41.7		
Mutant	5	5.3		
Unknown	78	53.0		

**Table 2:** Association of microbial DNA fragments between CRC patients and healthy subjects.

DNA	Gene Target	Detection	Patients	Healthy Individuals	*p*-Value
Microbial DNA fragments	DNA coding for 16S rRNA	Positive	57 (43.2%)	16 (16%)	<0.001
		Negative	75 (56.8%)	84 (84.0)	
	β-galactosidase gene of *E. coli*	Positive	27 (20.5%)	16 (16%)	0.387
		Negative	105 (79.5%)	84 (84%)	
	Glutamine synthase gene of *B. fragilis*	Positive	42 (31.8%)	0 (0%)	<0.001
		Negative	90 (68.2%)	100 (100%)	
	DNA coding for 5.8S rRNA of *C. albicans*	Positive	48 (36.4%)	0 (0%)	<0.001
		Negative	84 (63.6%)	100 (100%)	

**Table 3:** Association of Toll-like receptors (*TLRs*) and vitamin D receptors (*VDR*) between CRC patients and healthy subjects.

Polymorphism	Gene Target	Detection	Patients	Healthy Individuals	*p*-Value
*VDR* polymorphisms	*Taq*I	wild type	55 (41.7%)	71 (71%)	<0.001
		heterozygous	57 (43.2%)	26 (26%)	
		homozygous	20 (15.2%)	3 (3%)	
	*Apa*I	wild type	49 (37.1%)	52 (52%)	<0.001
		heterozygous	49 (37.1%)	40 (40%)	
		homozygous	34 (25.8%)	8 (8%)	
	*Fok*I	wild type	41 (31.1%)	55 (55%)	<0.001
		heterozygous	71 (53.8%)	40 (40%)	
		homozygous	20 (15.2%)	5 (5%)	
	*Bsm*I	wild type	49 (37.1%)	55 (55%)	<0.001
		heterozygous	66 (50%)	43 (43%)	
		homozygous	17 (12.9%)	2 (2%)	
*TLR* polymorphisms	*TLR4*—D299G	wild type	31 (23.5%)	100 (100%)	<0.001
		heterozygous	50 (37.9%)		
		homozygous	51 (38.6%)		
	*TLR4*—T399I	wild type	32 (24.2%)	100 (100%)	<0.001
		heterozygous	48 (|36.4%)		
		homozygous	52 (39.4%)		
	*TLR9*—T1237C	wild type	13 (9.8%)	52 (52%)	<0.001
		heterozygous	70 (53%)	48 (48%)	
		homozygous	49 (37.1%)		
	*TLR9*—T1486C	wild type	13 (9.8%)	52 (52%)	<0.001
		heterozygous	72 (54.5%)	48 (48%)	
		homozygous	47 (35.6%)		
	*TLR2*-196 to -174bp	ins/ins		100 (100%)	<0.001
		ins/del	57 (43.2%)		
		del/del	75 (56.8%)		

**Table 4:** Correlation between microbial DNA fragments, Toll-like receptor (*TLR*) and vitamin D receptor (*VDR*) polymorphisms parameters (values against each category represents *p*-values).

Target	*TLR*					*VDR*			
Gene Target	*TLR2*-196 to -174bp	*TLR4*—D299G	*TLR4*—T399I	*TLR9*—T1237C	*TLR9*—T1486C	*Taq*I	*Apa*I	*Fok*I	*Bsm*I
16S rRNA	0.172	0.009	0.043	0.549	0.567	<0.001	0.112	<0.001	0.2534
*Escherichia coli*	0.074	0.091	0.093	0.617	0.548	<0.001	0.003	0.0590	0.553
*Bacteroides fragilis*	0.522	0.025	0.087	0.229	0.258	<0.001	0.009	<0.001	0.075
*Candida albicans*	0.528	0.798	0.619	0.896	0.928	<0.001	0.015	0.027	0.029
*TLR2*-196 to -174bp		<0.001	<0.001	<0.001	<0.001	0.671	0.399	0.080	0.004
*TLR4*—D299G	<0.001		<0.001	<0.001	<0.001	0.043	<0.001	0.015	<0.001
*TLR4*—T399I	<0.001	<0.001		<0.001	<0.001	0.042	<0.001	0.036	<0.001
*TLR9*—T1237C	<0.001	<0.001	<0.001		<0.001	0.364	0.008	0.012	<0.001
*TLR9*—T1486C	<0.001	<0.001	<0.001	<0.001		0.423	0.018	0.029	<0.001
*Taq*I	0.671	0.043	0.042	0.364	0.423		0.395	<0.001	<0.001
*Apa*I	0.399	<0.001	<0.001	0.008	0.018	0.395		<0.001	<0.001
*Fok*I	0.080	0.015	0.036	0.012	0.027	<0.001	<0.001		<0.001
*Bsm*I	0.003	<0.001	<0.001	<0.001	<0.001	<0.001	<0.001	<0.001	

**Table 5:** Correlation of tumor mutational (*KRAS*/*NRAS*/*BRAF^V600E^*) and microsatellite instability (MSI) status with microbial DNA fragments, *TLR,* and *VDR* gene mutant alleles (values against each category represents *p*-values).

Mutation/ MSI Status	*TLR9*—T1237C	*TLR9*—T1486C	*Taq*I	*Apa*I	*Fok*I	*Bsm*I
*KRAS*	0.014	0.006				
*BRAF^V600E^*		0.045				
*MSI*	0.012		0.025	0.047	0.001	<0.001

**Table 6:** Univariate and multivariate Cox regression analysis for disease free (DFS) and overall (OS) survival.

Factor	Univariate				Multivariate			
	DFS		OS		DFS		OS	
Factor	HR (Range)	*p*-Value	HR (Range)	*p*-Value	HR (Range)	*p*-Value	HR (Range)	*p*-Value
*BRAF* mut vs. wt	17.05 (2.4–123.4)	0.005	-	-				
*B. fragilis* pos vs. neg	2.09 (1.0–4.3)	0.047	-	-	33.85 (1.8–622.4)	0.018	-	-
*C. albicans* pos vs. neg	-	-	3.57 (1.2–10.3)	0.019				
*VDR*—*Apa*I	1.56 (1.0–2.3)	0.031	-	-				
Histology adeno vs. mucinus	2.72 (1.1–6.8)	0.031	-	-				

## Data Availability

All relevant data are within the paper and its Supporting Information files.

## Supplementary Materials

The following supporting information can be downloaded at: https://www.mdpi.com/article/10.3390/cancers14184407/s1, Table S1: Raw data material.
